# Supplementary material for: Performance of Machine Learning Models in Predicting 30-Day General Medicine Readmissions Compared to Traditional Approaches in Australian Hospital Setting
Source: Healthcare (Basel). 2025 May 23;13(11):1223. doi: 10.3390/healthcare13111223 (PMC12153988; doi:10.3390/healthcare13111223)
Supplement: Supplementary file 1 [file healthcare-13-01223-s001.zip › Supplementary Table S2 .pdf]

Table S2 Proportion of variables with missing values

Variable	Missing	Total	Percent missing
HFRS	775	5371	14.43%
CRP	319	5371	5.94%
Race	113	5371	2.10%
Platelet count	72	5371	1.34%
Haemoglobin	62	5371	1.15%
WBC count	62	5371	1.15%
NLR	62	5371	1.15%
Creatinine	57	5371	1.06%
Sodium	57	5371	1.06%
Urea	57	5371	1.06%
Albumin	84	5371	1.56%
IRSD	11	5371	0.20%
Living status	32	5371	0.60%
Sex	1	5371	0.02%

HFRS, hospital frailty risk score; ; CRP, c-reactive protein WBC, white blood cell; NLR, neutrophil:lymphocyte ratio; NLR, neutrophil:lymphocyte ratio IRSD, index of relative socioeconomic disadvantage
