# Supplementary material for: Performance of Machine Learning Models in Predicting 30-Day General Medicine Readmissions Compared to Traditional Approaches in Australian Hospital Setting
Source: Healthcare (Basel). 2025 May 23;13(11):1223. doi: 10.3390/healthcare13111223 (PMC12153988; doi:10.3390/healthcare13111223)
Supplement: Supplementary file 1 [file healthcare-13-01223-s001.zip › Supplmentary Table S1.pdf]

Table S1. Variables used in prediction models

	Variable Name
**1**	Age
**2**	Sex
**3**	Race
**4**	HFRS
**5**	No of ED visits in previous 6months
**6**	Total number of hospital admission in past one year
**7**	Charlson index
**8**	Albumin
**9**	Creatinine
**10**	Haemoglobin
**11**	Sodium
**12**	WBC count
**13**	IRSD
**14**	CAD
**15**	CKD
**16**	Chronic Liver Disease
**17**	Smoking
**18**	Alcohol abuse
**19**	Hypertension
**20**	Congestive Heart Failure
**21**	Stroke
**22**	CRP
**23**	Urea
**24**	Discharge after hours
**25**	Diabetes
**26**	Chronic Lung Disease
**27**	Platelet count
**28**	Living status
**29**	NLR
**30**	Polypharmacy
**31**	Weekend discharge
**32**	LOS

HFRS, hospital frailty risk score; ED, emergency department; WBC, white blood cell; IRSD, index of relative socioeconomic disadvantage; CAD, coronary artery disease; CKD, chronic kidney disease; CRP, c-reactive protein; NLR, neutrophil:lymphocyte ratio; LOS, length of hospital stay
