# Supplementary figures and images for: Performance of Machine Learning Models in Predicting 30-Day General Medicine Readmissions Compared to Traditional Approaches in Australian Hospital Setting
Source: Healthcare (Basel). 2025 May 23;13(11):1223. doi: 10.3390/healthcare13111223 (PMC12153988; doi:10.3390/healthcare13111223)

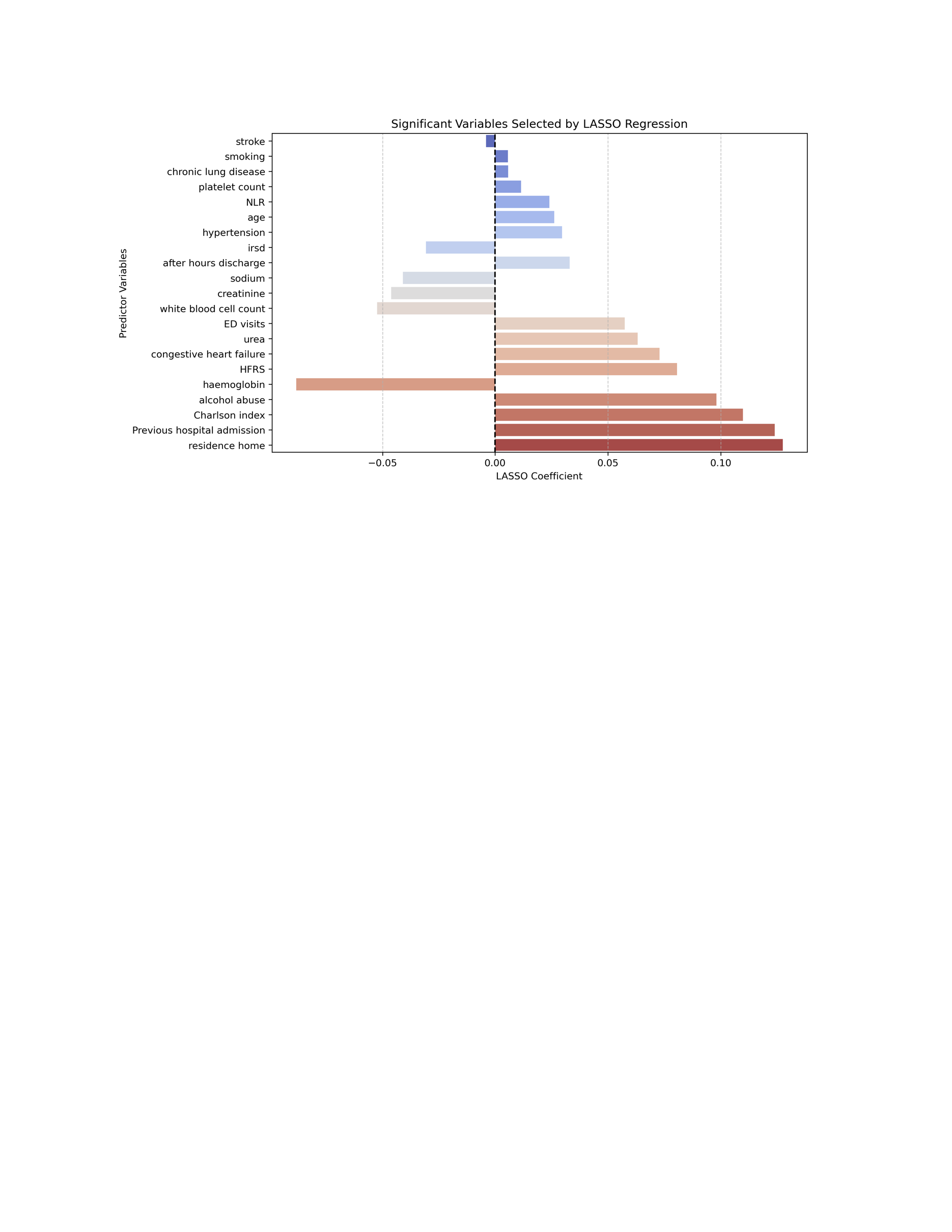

Supplement: Supplementary file 1 [file healthcare-13-01223-s001.zip › Figure S1.png]
